# Draft Genome Sequence of *Syntrophorhabdus aromaticivorans* Strain UI, a Mesophilic Aromatic Compound-Degrading Syntroph

**DOI:** 10.1128/genomeA.01064-13

**Published:** 2014-02-06

**Authors:** Masaru K. Nobu, Takashi Narihiro, Hideyuki Tamaki, Yan-Ling Qiu, Yuji Sekiguchi, Tanja Woyke, Lynne Goodwin, Karen W. Davenport, Yoichi Kamagata, Wen-Tso Liu

**Affiliations:** a Department of Civil and Environmental Engineering, University of Illinois at Urbana-Champaign, Urbana, Illinois, USA; b Bioproduction Research Institute, National Institute of Advanced Industrial Science and Technology (AIST), Tsukuba, Ibaraki, Japan; c Key Laboratory of Biofuels, Qingdao Institute of Bioenergy and Bioprocess Technology, Chinese Academy of Sciences, Qingdao, Shandong Province, People’s Republic of China; d Biomedical Research Institute, National Institute of Advanced Industrial Science and Technology (AIST), Tsukuba, Ibaraki, Japan; e DOE Joint Genome Institute, Walnut Creek, California, USA; f Los Alamos National Laboratory, Los Alamos, New Mexico, USA; g Bioproduction Research Institute, National Institute of Advanced Industrial Science and Technology (AIST), Sapporo, Hokkaido, Japan

## Abstract

*Syntrophorhabdus aromaticivorans* strain UI is a mesophilic bacterium capable of degrading aromatic substrates in syntrophic cooperation with a partner methanogen. The draft genome sequence is 3.7 Mb, with a G+C content of 52.0%.

## GENOME ANNOUNCEMENT

*Syntrophorhabdus aromaticivorans* strain UI is one of the few isolated bacteria capable of anaerobic degradation of aromatic compounds in syntrophic association with a methanogen (1). *S. aromaticivorans* UI was isolated from a mesophilic bioreactor treating aromatic compound-containing wastewater as a representative of the family *Syntrophorhabdaceae* of the class *Deltaproteobacteria*. Other isolates include members of the genus *Syntrophus* (*S. aciditrophicus* strain SB, *S. buswellii* strain DM-2, and *S. gentianae* strain HQGö1) of the class *Deltaproteobacteria* and members of the genera *Pelotomaculum* (*P. isophthalicum* strain JI and *P. terephthalicum* strain JT) and *Sporotomaculum* (*S. hydroxybenzoicum* strain BT and *S. syntrophicum* strain FB) of the class *Clostridia* (2–7). Of these aromatic compound-degrading syntrophs, strain UI is uniquely capable of degrading phenol (1). It is also known to syntrophically degrade *p*-cresol, 4-hydroxybenzoate, isophthalate, and benzoate with a hydrogenotrophic methanogen (1), suggesting that *Syntrophorhabdaceae* members likely play an important role in anaerobic treatment of industrial wastewater-containing aromatic compounds.

The draft genome of *S. aromaticivorans* strain UI was generated from a coculture of strain UI and *Methanospirillum hungatei* strain JF-1 at the DOE Joint Genome Institute (JGI) with a combined approach using Illumina (8) and 454 (9) technologies. We constructed and sequenced an Illumina GAII shotgun library (69,270,135 reads) totaling 5,264.5 Mb and a paired-end 454 library (322,727 reads, an average insert size of 8 kb) totaling 64 Mb of 454 data. Assemblies were performed using a combination of Newbler version 2.3-PreRelease-6/30/2009, Velvet version 1.0.13 (10), and parallel Phrap version SPS-4.24 (High Performance Software, LLC). The software Consed (11–13) was used in the following finishing process. Illumina data were used to correct potential base errors and increase consensus quality using the software Polisher developed at JGI (A. Lapidus, unpublished data). Possible misassemblies were corrected using gapResolution (C. Han, unpublished data) or DupFinisher (14) or sequencing cloned bridging PCR fragments with subcloning. Gaps between contigs were closed by editing in Consed, by PCR, and by bubble PCR (J.-F. Cheng, unpublished data) primer walks. A total of 308 additional reactions were necessary to close gaps and to raise the quality of the finished sequence. *M. hungatei* strain JF-1 sequences were removed using scaffold read coverage and BLAST. The estimated genome size is 3.7 Mb, and the final assembly is based on 30.3 Mb of 454 draft data and 5,235.1 Mb of Illumina draft data, which provide an average 8.2× coverage and 1,414.9× coverage of the genome, respectively.

The strain UI draft genome is composed of three linear scaffolds and has a G+C content of 52.0%. This genome contains 3,691 genes, 3,632 protein-coding and 59 RNA genes. The Integrated Microbial Genomes (IMG) pipeline (15) annotated 71.82% of the protein-coding genes with function prediction. The 59 RNA genes consisted of 3 rRNA genes (5S, 16S, and 23S), 51 tRNA genes, and 5 other RNA genes. We are currently exploring this genome to identify novel syntrophic aromatic degradation genes and pathways for further comparison with benzoate-degrading syntrophs.

### Nucleotide sequence accession numbers.

This whole-genome shotgun project has been deposited at DDBJ/EMBL/GenBank under the accession no. AJUN00000000. The version described in this paper is the first version, AJUN01000000.
